# Cytoarchitectonic Characterization and Functional Decoding of Four New Areas in the Human Lateral Orbitofrontal Cortex

**DOI:** 10.3389/fnana.2020.00002

**Published:** 2020-02-05

**Authors:** Magdalena Wojtasik, Sebastian Bludau, Simon B. Eickhoff, Hartmut Mohlberg, Fatma Gerboga, Svenja Caspers, Katrin Amunts

**Affiliations:** ^1^Cécile and Oskar Vogt—Institute for Brain Research, Medical Faculty, Heinrich-Heine-University Düsseldorf, Düsseldorf, Germany; ^2^Institute of Neuroscience and Medicine 1 (INM-1), Research Center Jülich, Jülich, Germany; ^3^Institute of Neuroscience and Medicine 7 (INM-7), Research Center Jülich, Jülich, Germany; ^4^Institut für Systemische Neurowissenschaften, Medizinische Fakultät, Heinrich-Heine Universität Düsseldorf, Düsseldorf, Germany; ^5^Institute for Anatomy I, Medical Faculty, Heinrich-Heine-University Düsseldorf, Düsseldorf, Germany

**Keywords:** lateral orbitofrontal cortex, BA47, human brain atlas, cytoarchitecture, maximum probability maps, meta-analytic connectivity modeling, JuBrain, BigBrain

## Abstract

A comprehensive concept of the biological basis of reward, social and emotional behavior, and language requires a deeper understanding of the microstructure and connectivity of the underlying brain regions. Such understanding could provide deeper insights into their role in functional networks, and form the anatomical basis of the functional segregation of this region as shown in recent *in vivo* imaging studies. Here, we investigated the cytoarchitecture of the lateral orbitofrontal cortex (lateral OFC) in serial histological sections of 10 human postmortem brains by image analysis and a statistically reproducible approach to detect borders between cortical areas. Profiles of the volume fraction of cell bodies were therefore extracted from digitized histological images, describing laminar changes from the layer I/layer II boundary to the white matter. As a result, four new areas, Fo4–7, were identified. Area Fo4 was mainly found in the anterior orbital gyrus (AOG), Fo5 anteriorly in the inferior frontal gyrus (IFG), Fo6 in the lateral orbital gyrus (LOG), and Fo7 in the lateral orbital sulcus. Areas differed in cortical thickness, abundance and size of pyramidal cells in layer III and degree of granularity in layer IV. A hierarchical cluster analysis was used to quantify cytoarchitectonic differences between them. The 3D-reconstructed areas were transformed into the single-subject template of the Montreal Neurological Institute (MNI), where probabilistic maps and a maximum probability map (MPM) were calculated as part of the JuBrain Cytoarchitectonic Atlas. These maps served as reference data to study the functional properties of the areas using the BrainMap database. The type of behavioral tasks that activated them was analyzed to get first insights of co-activation patterns of the lateral OFC and its contribution to cognitive networks. Meta-analytic connectivity modeling (MACM) showed that functional decoding revealed activation in gustatory perception in Fo4; reward and somesthetic perception in Fo5; semantic processing and pain perception in Fo6; and emotional processing and covert reading in Fo7. Together with existing maps of the JuBrain Cytoarchitectonic Atlas, the new maps can now be used as an open-source reference for neuroimaging studies, allowing to further decode brain function.

## Introduction

The lateral orbitofrontal cortex (lateral OFC) includes the cytoarchitectonically defined Brodmann area (BA) 47 (Brodmann, 1909). It seems to occupy a structurally variable part of the human brain (Chiavaras and Petrides, 2000). According to Brodmann, BA 47 spans over the lateral orbital gyrus (LOG) with extensions into the posterior parts of the ventrolateral frontal cortex and anterior parts of the inferior frontal gyrus (IFG; Brodmann, 1909). As known from previous studies, the macroanatomy itself is variable with respect to the sulcal and gyral patterns including interhemispheric differences (Chiavaras and Petrides, 2000; Chiavaras et al., 2001; Kringelbach and Rolls, 2004; Rodrigues et al., 2015; Rolls et al., 2015). In many cases, the OFC is composed of an “H”-shaped pattern of sulci, which is characterized by the lateral and medial orbital sulcus (LOS, MOS). They run parallel to each other, are separated by the anterior orbital gyrus (AOG), and connected through the transverse orbital sulcus (TOS). Other patterns have been described as well (Ono et al., 1990; Chiavaras et al., 2001; Rodrigues et al., 2015), but the relationship of the different sulcal patterns with the microstructure at the level of areas are not well understood.

**Figure 1:**
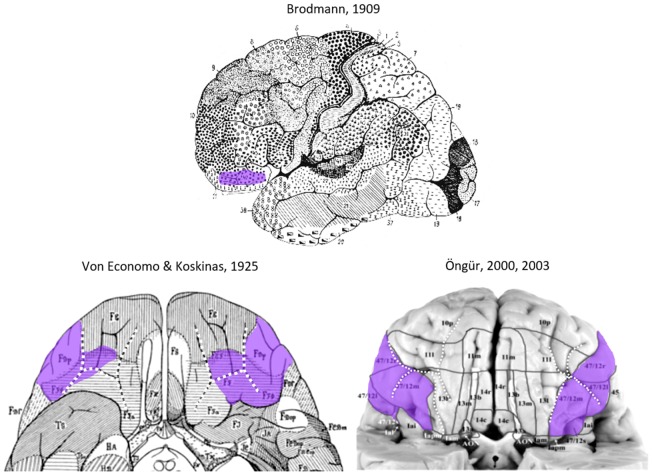
Area 47 in the lateral orbitofrontal cortex (lateral OFC) in the human brain according to previous cytoarchitectonical analyses(Brodmann, 1909; von Economo and Koskinas, 1925; Von Economo, 1929; Öngür et al., 2003). The sulcal pattern is highlighted with white dotted lines. Images have been modified such that the sulcal patterns are highlighted with white dotted lines, and the approximate extent of Area 47 is labeled in purple.

Previous studies have shown that the lateral OFC could be divided into a different number of areas. The parcellation schemes became more complex and fine-grained over time (see Figure 1). Different approaches were applied to map the lateral OFC including histological techniques such as cytoarchitectonic analysis or the function-driven approach using the functional magnetic resonance imaging (fMRI) technique. The varying number of areas, their size and location were influenced by the different analysis techniques as well as intersubject variability in brain shape and size.

In addition, different conceptual approaches were applied to characterize the lateral OFC, and introduced, for example, subareas and transitions. The term “area 47/12” was first *postulated by* Öngür and Price (2000). Further studies took up this term (Petrides and Pandya, 2002; Öngür et al., 2003; Kringelbach and Rolls, 2004; Deng et al., 2017), and assigned it to one large area surrounding the LOG. In combination with the medial OFC, the designations 47 m for the medial OFC and 47l for the lateral OFC were postulated (Uylings et al., 2000, 2010). Further terms were introduced, which reflected the topography, i.e., 47 m for the medial frontal cortex and 47° for the OFC (Neubert et al., 2015). Recently, some areas in the medial OFC have been named after their topographic location, abbreviated and numbered consecutively. Research from our own group identified area Fo1 in the anterior gyrus rectus, Fo2 in the posterior gyrus rectus, and area Fo3 reaching from the inner medial orbital sulcus to the outer medial orbital sulcus, enclosing Fo1 and Fo2 laterally (Henssen et al., 2016). The analysis was based on cytoarchitecture, and considered changes in cell density and cell distribution, mainly in layers III, IV and V.

Functional analyses of the lateral OFC revealed that this region is involved in the processing of gustatory, olfactory or somatosensory rewarding stimuli but also the processing of emotional punishments, evaluating and updating the emotional status, maintaining social behavior, active retrieval of information, semantic processing, verb generation, processing of stimuli that have a coherent temporal structure, music listening, assigning value to certain things and events and decision-making (Papathanassiou et al., 2000; Levitin and Menon, 2003; Kringelbach and Rolls, 2004; Petrides, 2005; Campbell-Meiklejohn et al., 2012; Alluri et al., 2013; Liu et al., 2015; Neubert et al., 2015; Rolls et al., 2015; Hirose et al., 2016). Tasks that require quick responses while retrieving information from the long-term and working memory also showed activations in the lateral OFC. This region seems to create an interconnecting role between the frontal cortex and the hippocampus (Ross et al., 2013; Deng et al., 2017; Rudebeck and Rich, 2018). Another functional study separated the OFC into a medial and a lateral portion (Zald et al., 2014). The latter corresponded to BA 47 and co-activated with areas in the IFG and area BA 46/9 of the dorsomedial frontal cortex along with several subcortical structures, e.g., the amygdala, hippocampus and nucleus accumbens among others.

The present study provides a comprehensive cytoarchitectonic analysis of the human lateral OFC using a computerized approach to detect cytoarchitectonic borders between adjacent areas based on image analysis and statistical criteria (Schleicher et al., 1998, 1999, 2005), and provides cytoarchitectonic probabilistic maps in 3D reference space (Amunts and Zilles, 2015). The maps have been created based on the same methods as used for previous mapping studies of our group [most recent include, e.g., the parietal cortex (Richter et al., 2019), the motor cortex (Ruan et al., 2018), and the fusiform gyrus (Lorenz et al., 2017)], and allow to integrate them into a coherent atlas framework of the human brain. Meta-analytic connectivity modeling (MACM) was conducted to assess all task-based functional connectivities between the lateral OFC areas and their respective co-activated cortical and subcortical brain regions in the same reference space. Their corresponding activation foci with the lateral OFC areas as seed regions were detected *via* the BrainMap database (Laird et al., 2009; Robinson et al., 2010). The present work aims to combine the structural peculiarity of the lateral OFC with its functional properties and to give a first insight into the cognitive networks in which the lateral OFC is integrated.

## Materials and Methods

### Processing of Postmortem Brains

Ten brains (five females with age range of 59–86 years, and five males with age range of 30–75 years, mean age of 65, 8 years) were obtained from the body donor program of the Department of Anatomy at the University Hospital Düsseldorf of the Heinrich-Heine-University in accordance with legal requirements with no indications of neurologic or psychiatric diseases in clinical records. The postmortem delay did not exceed 24–36 h (Table 1). The brains were fixed in 4% buffered formalin (pH 7.4) or Bodian’s fixative for at least 6 months. All brains underwent magnetic resonance imaging on a Siemens 1.5 Tesla scanner (Erlangen, Germany) using a T1-weighted 3D FLASH sequence (flip angle 40°, repetition time TR 40 ms, echo time TE 5 ms). Obtained images were used as an undistorted spatial reference for the 3D-reconstruction of the histological sections as previously described (Bludau et al., 2014).

**Table 1:** List of postmortem brains used for cytoarchitectonic analysis.

Brain ID	Gender	Cause of death
pm1	Female	Bladder carcinoma
pm4	Male	Rectal cancer
pm5	Female	Cardiorespiratory insufficiency
pm8	Female	Kidney failure
pm9	Female	Generalized atherosclerosis, aortic valve stenosis, left heart insufficiency, basal ganglia infarction
pm11	Male	Heart attack
pm13	Male	Drowning
pm14	Female	Cardiorespiratory insufficiency, right-sided breast cancer
pm20	Male	Decompensated heart failure, respiratory insufficiency, prostate cancer, tumor anemia
pm21	Male	Bronchopneumonia, recurrence of Hodgkin’s disease, deep vein thrombosis

Brains were embedded in paraffin and serially sectioned in the coronal plane on a large-scale microtome (thickness of 20 μm). Every 15th section (corresponding to a distance of 300 μm) was mounted on a glass slide covered with gelatin, stained for cell bodies using a silver staining technique (Merker, 1983), and digitized on a flatbed scanner (resolution of 1,200 dpi). At least every 60th section was analyzed (distance between them of 1.200 μm).

### Identification of Cytoarchitectonic Borders Based on the Grey Level Index (GLI)

The identification of cytoarchitectonic borders (Supplementary Figure S1A) was based on image analyses of rectangular regions of interests (ROIs) in every 60th histological section, and statistical criteria (Schleicher et al., 1998, 1999, 2005). ROIs were digitized with a CCD camera (Axiocam MRm, ZEISS, Germany), which was connected to a computer-controlled optical light microscope with motorized scanning stage (Axioplan 2 imaging, ZEISS, Germany). The Zeiss image analysis software Axiovision (version 4.6) allowed to scan the defined ROIs in a mosaic-like way with an in-plane resolution of 1.02 μm per pixel (Supplementary Figure S1B). GLI images were computed in adjacent square fields of 17 × 17 μm using in-house written MatLab scripts (The MathWorks, Inc., Natick, MA, USA) as a robust estimate of the volume fraction of cell bodies (Wree et al., 1982; Schleicher et al., 1986, 2005; Supplementary Figure S1C). The GLI is defined as an estimate of the local volume density of cellular structures that is influenced by section thickness (here 20 μm for all sections). The GLI measures the areal proportion as a numerical equivalent for the volume density (Schleicher et al., 1986). The GLI is the ratio of the area covered by image elements, which are darker than a given gray value threshold, to the entire area of the measuring field, which is of fixed size (Schleicher et al., 1986). The gray value threshold was set to the gray value of the boundary between the dark cellular image elements and the bright background by analyzing the gray value histogram of the image (Schleicher et al., 1986). The cortical ribbon was delineated by an outer contour (border between layer I and II) and an inner contour line (border between layer VI and white matter; Supplementary Figure S1D). Curvilinear traverses running perpendicular to the cortical layers from the outer to the inner contour were defined to calculate GLI profiles (Supplementary Figure S1E), and GLI values, reflecting the laminar changes in cytoarchitecture, were extracted along the traverses. GLI profiles were described by a 10-element feature vector consisting of the mean GLI value, the center of gravity in x- and y-direction, the standard deviation, kurtosis, skewness and the equivalent parameters of the profiles’ first derivatives (Schleicher et al., 2005). Vectors of each profile were used to calculate the Mahalanobis distance (MD, Mahalanobis et al., 1949) between blocks of profiles. The MD is a measure for cytoarchitectonic dissimilarity between profiles. The larger the dissimilarity, the higher the MD, and vice versa. A Hotelling’s T2 test with Bonferroni correction was applied to test for significance between differences of profiles. A predefined number of profiles was combined into a block, with a block size ranging from 12 to 30 profiles to increase robustness of the procedure. MDs were computed in a sliding window procedure for each profile position and every block size surrounding this position across the whole cortex in each ROI. If the MD reached a significant maximum at different block sizes at a certain profile position (Supplementary Figure S1F), a cytoarchitectonic border was assumed (Supplementary Figures S1G,H). Resulting areas were manually delineated in digitized high-resolution scans *via* the Section Tracer Online Tool developed in-house (Supplementary Figure S1I).

### Hierarchical Cluster Analysis of Cytoarchitectonic Dissimilarities Between Cortical Areas

A hierarchical cluster analysis was performed to detect structural dissimilarities between the lateral OFC areas, and compared to the adjacent areas Fo3 (Henssen et al., 2016), Fp1 (Bludau et al., 2014) as well as area 45 as part of Broca’s region (Amunts et al., 2004). Therefore, 15–20 consecutive profiles were extracted in three successive sections per area and hemisphere in each of the 10 brains at cortical locations where curvature and tangency had their lowest expanse. Each profile was represented by the 10-element feature vector, which enabled the analysis of linkage (Ward’s method) and distance (Euclidean distance) between given areas to quantify their degree of dissimilarity. The Euclidean distance describes the distances between pairs of neighboring profiles, i.e., the differences in the shape of these profiles, without taking into account the variability within clusters of profiles (Schleicher et al., 1998, 1999). An in-house written script for MatLab (The MathWorks, Inc., Natick, MA, USA) was used for the calculation. A value of a high Euclidean distance indicated a low structural similarity (and a large degree of cytoarchitectonic difference), and vice versa, a low value indicated high similarity. Respective brains were pooled either by gender (male/female) or by hemisphere (left/right). A dendrogram visualized the hierarchical clustering of all analyzed cortical areas.

### Probabilistic Cytoarchitectonic Maps in Stereotaxic Space and Maximum Probability Maps (MPMs)

The contour lines of the areas of the lateral OFC of all 10 brains were interactively traced onto 1,200 dpi high-resolution images of the histological sections, and 3D-reconstructed (Bludau et al., 2014). Spatially normalized areas of all 10 brains were transferred onto the T1-weighted, single-subject brain template of the Montreal Neurological Institute (MNI) “Colin27”. This brain template was used as the anatomical reference brain (Holmes et al., 1998; Evans et al., 2012) and transferred to anatomical MNI space (Amunts et al., 2005). After the superimposition of the areas in reference space, probabilistic maps were calculated. They showed the percentage of location and size probability of a given area in each voxel in the reference brain, and were color-coded values from 10% (blue) to 100% (red). Subsequently, a maximum probability map (MPM) was calculated for the whole lateral OFC, where each voxel was assigned to the cytoarchitectonic area with the highest locational probability in this voxel (Eickhoff et al., 2005). At borders of lateral OFC areas with unmapped or currently unknown areas (posteriorly adjacent cortex), the threshold for including a voxel into the MPM of each area was set to 0.4, resulting in a probability of 40% for each voxel to be assigned to a specific area (Eickhoff et al., 2005). The respective areal representations can be accessed and are available in the JuBrain Cytoarchitectonic Atlas^1^ as well as the new BigBrain template of the HBP atlas^2^, resembling an ultrahigh-resolution three-dimensional model of a human brain at nearly cellular resolution of 20 × 20 μm (Amunts et al., 2013) and the MNI template of the HBP human brain atlas^3^. In order to compare the data sets in the different template spaces, vector fields have been calculated based on a 400 μm isotropic down-sampled volume, to define a homeomorphic transformation between the BigBrain and the MNI space (Amunts et al., 2013).

### Volumetric Analysis

Individual shrinkage factors were obtained for each postmortem brain (Amunts et al., 2007). The ratio of the fresh brain volume was therefore divided by its volume after histological processing, further multiplied with the mean specific density of 1.033 g/mm^3^ (Zilles et al., 1988). Volume correction was obtained by calculation of areal proportions in each brain to enable the comparison between all brains due to their differing weight. A contrasting estimate was calculated between the means of grouped gender and hemispheres using in-house software written in MatLab (The MathWorks, Inc., Natick, MA, USA) as well as pair-wise permutation tests to detect significant differences of the volume proportion between the lateral OFC areas. The null distribution was estimated using Monte-Carlo simulation with a repetition of 1,000,000 iterations. The difference between all four areas was considered significant if the contrast estimate of the comparison exceeded 95% of the values under random distribution (*P* < 0.05).

### Analysis of Macro Anatomical Pattern in an Extended Sample of Brains

Considering the significant intersubject variability in the sulcal and gyral pattern of the lateral OFC, we investigated the individual macroanatomy of the OFC on images of the basal and lateral views of 26 human postmortem brains of the JuBrain Cytoarchitectonic Atlas, which were used in the past years for mapping (for an overview, see Amunts and Zilles, 2015). The 10 postmortem brains used for the cytoarchitectonic mapping and analysis were part of this sample. Previous studies (Ono et al., 1990; Chiavaras et al., 2001; Rodrigues et al., 2015) proposed three or four different sulcal patterns, which we applied. This resulted in a classification of four types of patterns of sulci and gyri in the 52 hemispheres. The paths of the respective sulci of the orbitofrontal cortex (OFC) were traced in the histological sections of the brains and labeled in the ventral views of the images of the postmortem brains.

### Functional Decoding of Areas in the Lateral OFC

The BrainMap^4^ database includes a large number of search criteria to limit the exploration for matching studies. On the experimental level, behavioral domains (BDs) and paradigm classes (PCs) enabled the specification of given experiments and the functional decoding of examined VOIs. Functional decoding of a given seed region involved the acquisition of all detectable functions by the over-representation of BDs and PCs in the experiments activating each VOI relative to the BrainMap database (Eickhoff et al., 2011). Studies with functional imaging data showing peak x-y-z-coordinates were explored using the following search criteria: normal mapping, activations only, using either fMRI or PET studies and only healthy subjects were included (Laird et al., 2011). This approach yielded a total number of 1,167 functional neuroimaging experiments at the time of analysis (Supplementary Table S1). No preselection of taxonomic categories had been conducted.

Functional characterization using BrainMap’s metadata was visualized with bar graphs displaying all BDs and PCs for every examined VOI with their respective probability likelihood ratio, indicating activation in each area by a particular BD and PC using forward and reverse inference. The former approach describes the probability of the observation of activity in a brain region, given the knowledge of the psychological process, whereas reverse inference defines the probability of a psychological process being present, given the knowledge of activation in a particular brain region. The respective bar graphs were transformed into network diagrams.

### Meta-analytic Connectivity Modeling on the Lateral OFC Areas

MACM was performed using the activation likelihood estimation (ALE) algorithm. It identified coincided whole-brain co-activation patterns from the neuroimaging study contingent of the BrainMap database as peak x-y-z coordinates in stereotaxic space for each lateral OFC seed region. Here, similarities in co-activation profiles of each VOI (Eickhoff et al., 2011) were extracted as activation foci from all matching neuroimaging studies showing potential functional co-activation and were displayed on the MNI ICBM 152 brain template. The MNI ICBM 152 reference space consists of brain scans from 152 different subjects. They were non-linearly registered in the MNI ICBM 152 coordinate system, and averaged. To establish a null-distribution reflecting a random spatial association between experiments, 10,000 permutations were calculated. All analyses were thresholded at an FWE-corrected threshold of *P* < 0.05 using the cluster-level FWE thresholding (Eickhoff et al., 2016).

In this context, we performed a conjunction analysis in which overlap of all four MACM co-activation maps was conducted. This allowed us to find out which brain regions were affected by all four lateral OFC areas. Additionally, contrast analyses were performed on each of the co-activation maps of the four lateral OFC areas with two contrasting co-activation patterns at a time to demonstrate any differing functional connectivity between the two respective seed VOIs and to identify unique functions for each area per hemisphere (Eickhoff et al., 2011).

## Results

### Cytoarchitecture of Areas Fo4–Fo7

**Figure 2:**
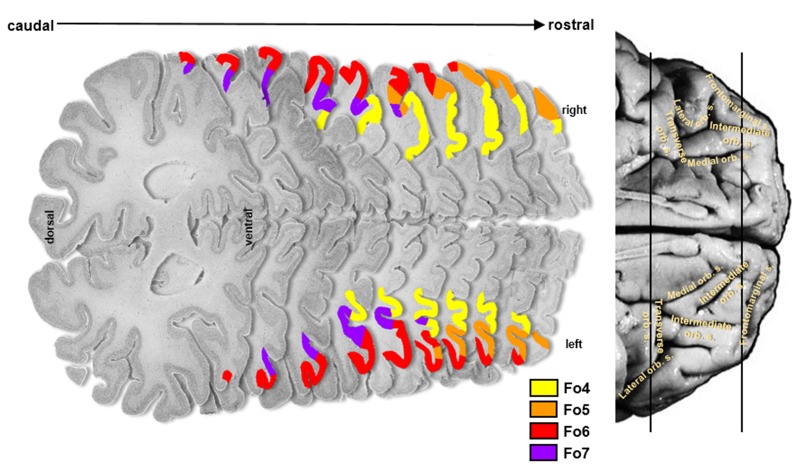
Localization of cytoarchitectonical areas Fo4, Fo5, Fo6, and Fo7 in the lateral OFC in serial histological sections of brain pm8.

The cytoarchitectonic analysis of the lateral OFC revealed four new areas (Figure 2): Fo4 was located at the AOG, lateral to area Fo3, which occupied the medial orbital gyrus and sulcus. Area Fo5 was spreading over the most anterior tip of the IFG, posterolateral to the frontomarginal sulcus (FMS) and ventrolateral to frontal polar area Fp1 (Bludau et al., 2014). Fo6 was encompassing the LOG following Fo5 posteriorly, and Fo7 was mostly occupying the lateral orbital sulcus and gyrus medial to Fo6, following Fo4 posteriorly; it did not exceed the TOS.

**Figure 3:**
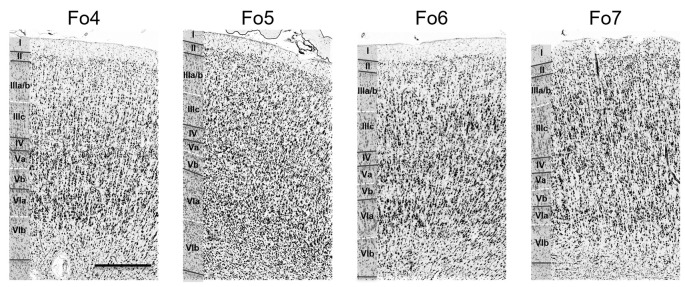
Cytoarchitecture of areas Fo4–Fo7 in the lateral OFC. Roman numerals indicate cortical layer I to VI. Scale bar = 500 μm.

Each area had a specific cytoarchitecture. Layer II of area Fo4 (Figure 3) was thin and loosely occupied by granule cells. Layer III was broad as compared to the other layers, and loosely packed by pyramidal cells, especially in layer IIIa and IIIb. In addition, all cells showed a relatively uniform size, except for sublayer IIIc showing slightly larger pyramidal cells. The thin layer IV was almost dysgranular. Layer V showed rather small pyramidal cells, but bigger than in sublayer IIIc. It was subdivided into sublayers Va and Vb. Cells in sublayer VIa were bigger and more densely packed than in sublayer VIb. Layer VI showed a rather smooth transition to the white matter.

Layer II of area Fo5 was also thin but tended to be thicker than in Fo4, and more cell dense. Sublayers IIIa and b displayed small-sized pyramidal cells with an increased cell size towards sublayer IIIc (Figure 3). Layer III showed a moderate cell density with lower amounts of cells than in the adjacent layers. Layer IV was also thin, but broader than in Fo4 and more populated with granule cells. Sublayer Va contained some large pyramidal cells, and sublayer Vb was more densely occupied than the analogs sublayer in area Fo4 (Figure 3). Sublayer VIa showed a higher cell density than layer V. The cortex-white matter border was also not sharp.

Compared to area Fo5, area Fo6 (Figure 3) had a slightly broader, but less cell-dense layer II with no sharp border to sublayer IIIa. Layer III was also less cell-dense. It showed medium-sized pyramidal cells in sublayer IIIc, and a decreasing cell size going towards the outer layers IIIb and IIIa. In addition, it contained larger pyramidal cells than in layer III of Fo5. Layer IV was wider and better visible than in areas Fo4 and Fo5, and more densely packed than Fo4, but not Fo5. Sublayer Va revealed prominent pyramidal cells, close to layer IV. Sublayer Vb showed a lower cell density than in Fo4 and Fo5. Similar to Fo4, sublayer VIa of area Fo6 was more densely packed with cells, but, in contrast to Fo4, showed a clear cut border to the white matter.

Area Fo7 (Figure 3) showed a more pronounced laminar pattern than the other three areas. Fo7 was characterized by large pyramidal cells in layer IIIc and a high cell density. Layer II was broad and contained uniformly sized granule cells. It was followed by a broad and densely packed layer III. Sublayer IIIc contained many large pyramidal cells and also smaller cells in IIIa and b. The broad and cell dense layer IV was also entangled with cells from sublayers IIIc and Va. Sublayer Va also showed large pyramidal cells, but smaller than those of IIIc. Sublayer Vb was the least cell dense. As in area Fo6, the transition between cortex and white matter was also clear-cut.

**Figure 4:**
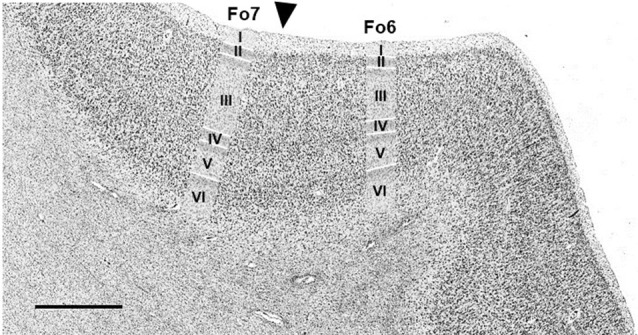
Cytoarchitectonic border of Fo6 with Fo7 (black arrowhead). Scale bar = 1 mm.

An example of a cytoarchitectonic border between Fo6 and Fo7 is shown in Figure 4. Compared to Fo6, Fo7 was characterized by a wide and cell dense layer III. In addition, the internal granular and pyramidal layers were accentuated by higher cell densities and cortex width. The thickness of layer VI appeared to be narrower in Fo7 than Fo6 in cortical regions with the comparative angle of sectioning. At the same time, it was more cell dense in sublayer VIa and exhibited an even better visible border to the white matter in sublayer VIb than Fo6.

The anterior ramus of the horizontal fissure formed a macroscopical landmark of Fo6 to the adjacent area 46. Posteromedial parts of the LOG and the entire lateral orbital sulcus were inhabited by Fo7, which was following Fo4 on the AOG, in few cases separated by an intermediate sulcus, ending in the TOS and slowly being cornered medially and posteriorly by new areas of the posterolateral orbital region which are still to be delineated.

### Differentiation of Areas Fo4–Fo7 From Neighboring Areas

The direct neighboring areas were frontopolar area Fp1 (Bludau et al., 2014) at the lateral surface of the frontal pole, anteriorly to Fo4 and Fo5, and Fo3 (Henssen et al., 2016) in the medial OFC. Fo3 bordered medially to Fo4. Additional orbitofrontal areas were located posteriorly to Fo6 and Fo7, but their cytoarchitecture has not yet been analyzed in detail. Area 46 was found lateral to area Fo6, beyond the horizontal ramus of the lateral fissure (Rajkowska and Goldman-Rakic, 1995).

**Figure 5:**
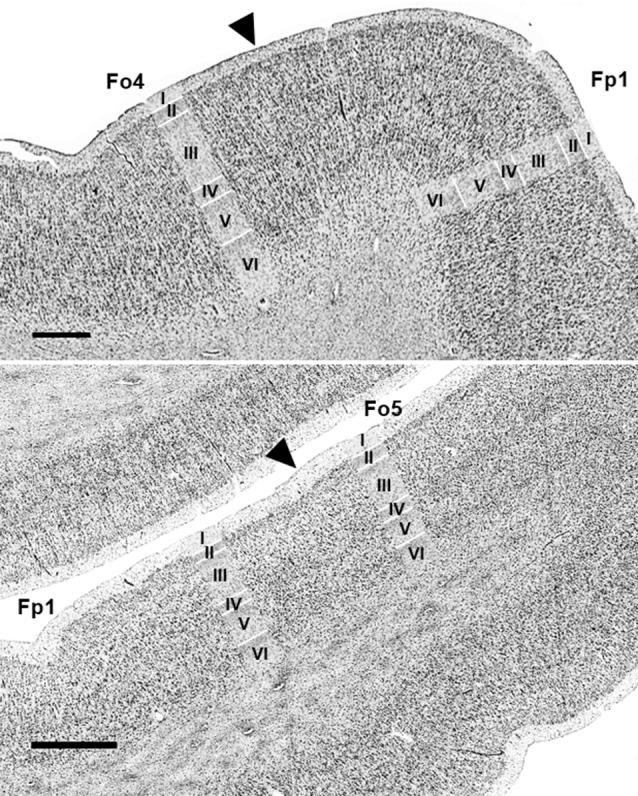
Cytoarchitectonic borders of Fp1 with Fo4 (upper panel) and Fp1 with Fo5 (lower panel). Arrowheads indicate borders between areas. Scale bars = 1 mm. Images come from the BigBrain, a high-resolution whole-brain model of the human brain, which can be found at: https://bigbrain.humanbrainproject.org/ (Amunts et al., 2013).

In most hemispheres, borders between areas Fp1 (Bludau et al., 2014) and Fo4 were found at the onset of the AOG, where Fo4 was following Fp1 posteriorly and was being medially cornered by Fo3 and laterally by Fo5 (Figure 5, upper panel). No clear macroscopical landmark was found between Fp1 and Fo4. Fo5 was located basolaterally to Fp1. Both areas shared borders in the most frontal part of the IFG right below the FMS (Figure 5, lower panel), which served as a macroscopical landmark separating both areas.

**Figure 6:**
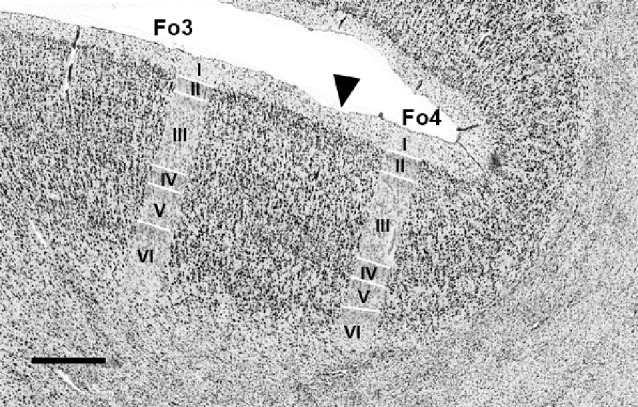
Cytoarchitectonic border of Fo3 with Fo4 (indicated by black arrowhead). Scale bar = 1 mm.

The medial orbital sulcus was associated as the macroscopical landmark between Fo3 (Henssen et al., 2016) and Fo4, with the latter area being located laterally to the sulcus. Borders between all cortical layers were better distinguishable in Fo4 than Fo3 (Figure 6).

The cytoarchitecture of these areas is summarized in Table 2. All areas were clearly separable from each other and exhibited distinct cytoarchitectonic characteristics representing true structural differences.

**Table 2:** Cytoarchitectonic characteristics of areas Fo4–Fo7 in the lateral orbitofrontal cortex (lateral OFC) and neighboring areas.

Area	Cytoarchitectonic characteristics
Fp1	Sharp border between layers I, II and III
	Dense layers II and IIIc
	Considerably larger pyramids in deeper than in upper layer III
	Broader layer IV than Fp2
Fo3	Large pyramidal cells in layer IIIc
	Inner granular layer with higher cell density and more prominent in its anterior than posterior part
	Layer Va of Fo3 with higher cell density than the respective layer of Fo4
Fo4	Narrow cortex throughout the area
	Indistinct borders between layers II, III, IV and V
	Uniformly packed layer III
	Middle-sized pyramidal cells in layer Va
Fo5	Very large pyramidal cells in densely packed layer IIIc
	Broad layer III
	More dense layer IV and V than Fo4
Fo6	Decreasing cell size in layer III from deeper to upper part
	Broad and cell-dense layer IV
	Middle-sized pyramidal cells in layer Va
Fo7	All layers very densely packed
	Indistinct borders between layers II, III, IV and V
	Large pyramidal cells in a deeper part of broad layer III
	Broad layer II, IV and V
	Layer IV more cell dense than Fo4–Fo6

*Descriptions for Fp1 and Fo3 adapted from Bludau et al. (2014) and Henssen et al. (2016), respectively*.

### Hierarchical Cluster Analysis of Cytoarchitectonic Differences and Similarities in the Lateral OFC

**Figure 7:**
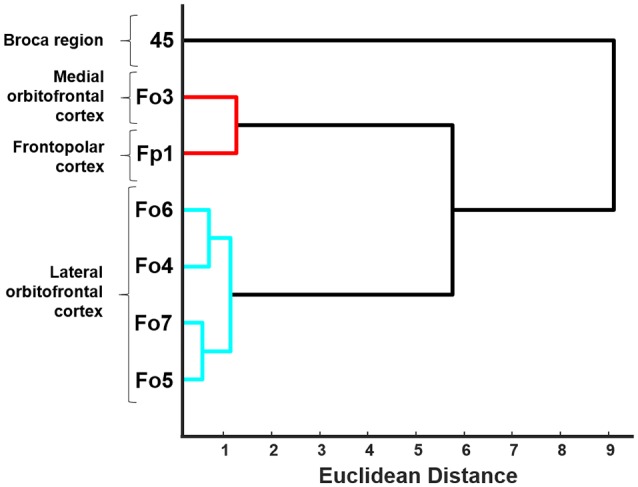
Dendrogram of the hierarchical cluster analysis of areas Fo4–Fo7 of the lateral OFC, area Fo3 of the medial OFC (Henssen et al., 2016), area Fp1 of the frontal pole (Bludau et al., 2014) and area 45 of the Broca region (Amunts et al., 2004). Euclidean distance was used as an indicator of structural dissimilarity. Areas of the lateral OFC are building a distinguishably separate cluster and show structural differences compared to their neighboring adjacent areas.

The hierarchical cluster analysis of areas Fp1, Fo3, Fo4, Fo5, Fo6, Fo7 and area 45 as an additional area from the ventral prefrontal cortex revealed a twofold clustering of areas with area 45 of the IFG on one branch, and all the other areas on the second branch (Figure 7). Fp1 and Fo3 were separated from the four lateral OFC areas on a higher hierarchical level. Fo4 and Fo6 were structurally more similar to each other than to Fo5 and Fo7, which was already visible in the cytoarchitectonic analysis. Fo5 and Fo7 differed from Fo4 and Fo6 by a denser cortex and larger cells in sublayer IIIc. The areas of the lateral OFC showed rather low degrees of structural dissimilarity. An increased degree of structural dissimilarity was found between the lateral OFC areas and Fo3 and Fp1, respectively. Area 45 was quite different from the areas of the lateral OFC, mostly due to its very large pyramidal cells in layer IIIc, not found in any other area adjacent to the lateral OFC.

### Cytoarchitectonic 3D-Maps and Intersubject Variability in Space

**Figure 8:**
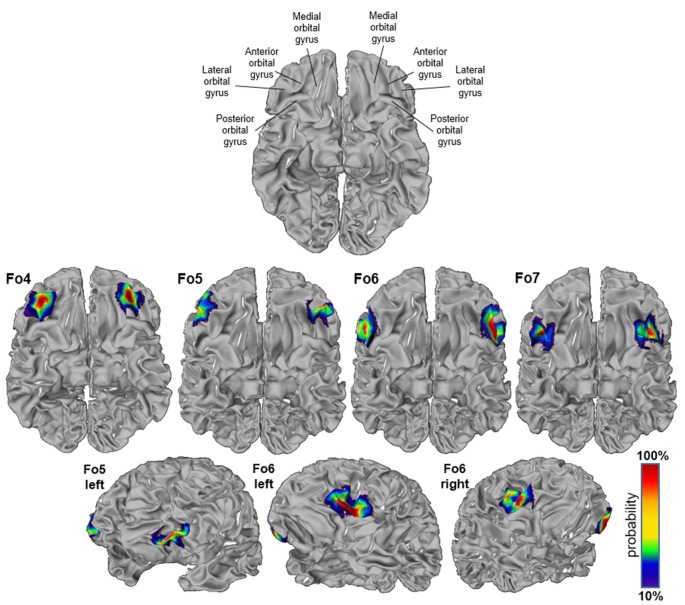
Probability maps registered to the anatomical Montreal Neurological Institute (MNI) anatomical reference brain “Colin27”; inflated version (JuBrain atlas: https://jubrain.fz-juelich.de/apps/cytoviewer/cytoviewer.php. Ventral views of areas Fo4–Fo7 (upper panels) and lateral views of Fo5 and Fo6 (lower panels). The probability is color-coded (red = full overlap of all 10 brains, dark blue = only one brain).

The intersubject variability of the four lateral OFC areas was quantified, and shown as probabilistic maps in the anatomical MNI reference space (Figure 8). An inflated version of the JuBrain Cytoarchitectonic Atlas visualized the areas in the depths of the sulci. Fo4 occupied the AOG in all 10 brains (red area in the center). Fo5 was predominantly present in the most anterior part of the IFG and also reached into the lateral orbital sulcus and the FMS. Fo6 occupied the LOG and its medially adjacent lateral orbital sulcus with a rather low probability of being present in the horizontal ramus of the lateral sulcus. Fo7 was likely to be found in the lateral orbital sulcus, also inhabiting the medial half of the LOG, as well as the posterior part of the medial orbital sulcus. The coordinates of all analyzed areas were provided in Table 3 for the MNI reference spaces MNI Colin27 and MNI ICBM 152.

**Table 3:** Center of gravity coordinates in MNI ICBM 152 space (upper panel) and anatomical MNI Colin27 space of continuous probability maps (lower panel) of all lateral OFC areas separated by hemisphere.

Area	Hemisphere	X	Y	Z
		Sagittal	Coronal	Horizontal
Center of gravity coordinates in MNI ICBM 152 space				
Fo4	Left	−28	53	−17
	Right	32	51	−16
Fo5	Left	−38	59	−10
	Right	44	55	−8
Fo6	Left	−45	39	−19
	Right	49	43	−15
Fo7	Left	−35	36	−10
	Right	38	37	−13
Center of gravity coordinates in anatomical MNI Colin 27 space of continuous probability maps				
Fo4	Left	−29	53	−10
	Right	32	48	−11
Fo5	Left	−37	56	−4
	Right	44	52	−1
Fo6	Left	−46	37	−11
	Right	49	37	−7
Fo7	Left	−35	34	−5
	Right	39	34	−8

**Figure 9:**
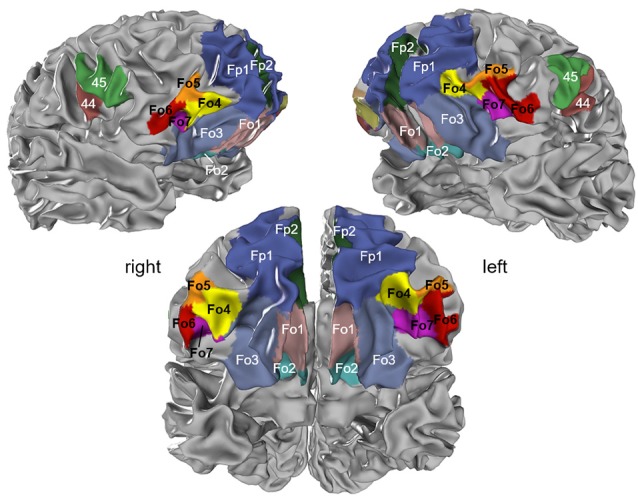
Maximum probability map (MPM) of the four areas registered to the single subject MNI template “Colin27”; view of right (upper left panel) and left (upper right panel) hemispheres and basal view (lower panel) showing the non-overlapping surface representations of the respective areas. Maps can be viewed at http://www.fz-juelich.de/inm/inm-1/jubrain_cytoviewer, and are available at https://bigbrain.humanbrainproject.org/ for download.

The MPM of all four lateral OFC areas and their respective neighbors represented a non-overlapping portrayal of the occupying surface representations. Surface representations of the MPM showed the extent of the areas with respect to gyri and sulci (Figure 9). In addition, cytoarchitectonically delineated areas neighboring the lateral OFC, i.e., Fp1 and Fo3, as well as Fo1, Fo2, area 44 and area 45 were displayed.

The new maps are available and open for download at https://bigbrain.humanbrainproject.org/ using the DOIs: 10.25493/29G0-66F (for Fo4), 10.25493/HJMY-ZZP (for Fo5), 10.25493/34Q4-H62 (for Fo6), and 10.25493/3WEV-561 (for Fo7), and are free to share and adapt under the creative commons license agreement.

### Volumes of Areas in the Lateral OFC

The volume of Fo5 was the smallest in this region, followed by Fo7, Fo4, and Fo6 as the largest area (Supplementary Table S2). The combined cortical volume of all lateral OFC areas for the left hemisphere was 3,111 ± 577 mm^3^, and for the right hemisphere 3,126 ± 549 mm^3^. Female brains had a volume of 3,075 ± 335 mm^3^ in the left, and 3,133 ± 237 mm^3^ in the right hemisphere. Male brains had a volume of 3,147 ± 796 mm^3^ in the left, and 3,119 ± 788 mm^3^ in the right hemisphere. All areal volumes were calculated for the shrinkage corrected brain volumes in each hemisphere. Using a permutation test, neither significant differences between the female and male brains, or left and right hemispheres were detected. The total cortical volume of all lateral OFC areas combined was 6,208 ± 492 mm^3^ in females, and 6,268 ± 1,576 mm^3^ in males. In both genders, the proportional distribution of left and right hemispheric lateral OFC was 49.5% and 50.5%, respectively, in females as well as 50.3% and 49.8%, respectively, in males.

### Macroanatomical Patterns and Their Variability

**Figure 10:**
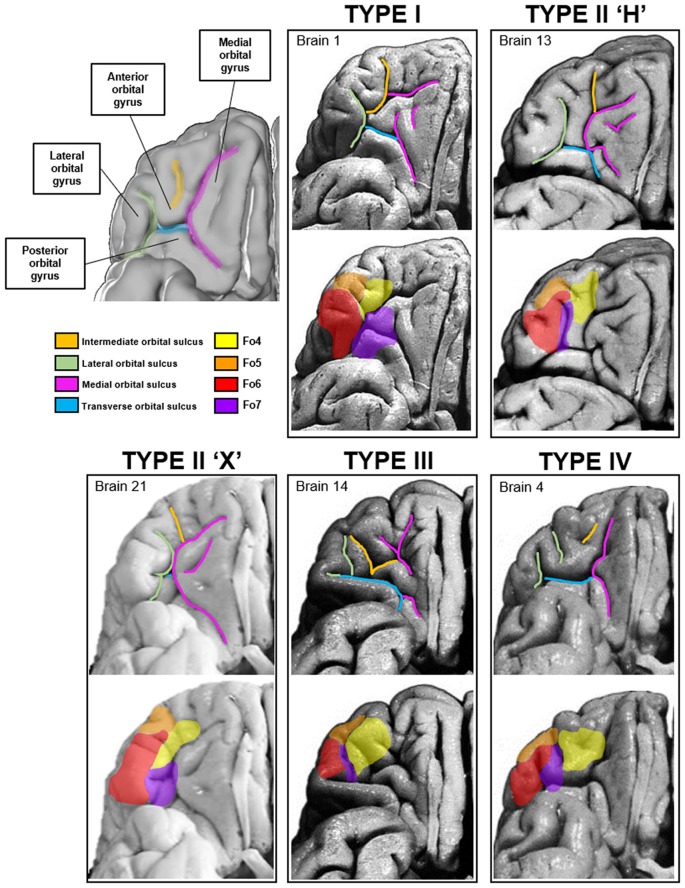
Four types of sulci and gyri patterns in the medial and lateral orbitofrontal region of the right hemisphere have been described according toOno, Chiavaras and Rodrigues (Ono et al., 1990; Chiavaras and Petrides, 2000; Chiavaras et al., 2001; Rodrigues et al., 2015). Localization of the respective areas differed between the four types according to the sulcal arrangements.

The macroanatomy of the OFC included a variety of different patterns of gyri and sulci. The different patterns were investigated on images of the basal views of the 52 hemispheres. Four different sulcal pattern types (one with two subtypes) were found according to the *criteria of* Chiavaras and Petrides (2000) and Chiavaras et al. (2001) as well as Rodrigues et al. (2015; Figure 10). Sulcal patterns were rather similar between the hemispheres in 13 out of 26 brains. The other half showed more pronounced interhemispheric differences in sulcus patterns. Interhemispheric differences were found in the anterior and posterior orbital gyrus and the medial and LOG, respectively, based on the formations of the medial, lateral and TOS.

The first type of pattern was formed by a segmented medial orbital sulcus, which consisted of a rostral and a caudal portion (Figure 10, Type I). The lateral orbital sulcus formed one single segment, and the TOS connected the caudal portions of the medial and lateral orbital sulcus. Of the 52 hemispheres, four left and nine right hemispheres distributed this pattern type (see Supplementary Figure S3).

The second pattern type could be divided into two different subtypes. The first subtype resembled the shape of the letter “H”. The medial and lateral orbital sulcus formed the two vertical “legs” and the TOS formed the connection between them. The AOG was separated from the posterior orbital gyrus (Figure 10, Type II). Additionally, an “X ”-pattern subtype was found. It was characterized by a short and barely segmented AOG. The medial orbital gyrus was touching the LOG and a barely visible TOS connected the caudal portions of the former two sulci. In the more pronounced H-pattern the TOS was still relatively short, while in the X-pattern it almost completely disappeared. From all 52 hemispheres, 16 revealed the H-subtype in the left and 8 in the right hemisphere. Additionally, three out of 52 hemispheres showed the X-subtype in the left and right hemispheres, respectively.

The third pattern was characterized by a prominent TOS dividing the posterior orbital gyrus from all other gyri (Figure 10, Type III). Both, the medial and lateral orbital sulcus were separated into rostral and caudal parts and only the caudal sulci were connected through the TOS. Out of all examined hemispheres, only one demonstrated this type in the left and four in the right hemisphere.

The fourth pattern type was characterized by a continuous medial orbital sulcus and a fragmented lateral orbital sulcus, the counterpart of the first pattern type so to speak (Figure 10, Type IV). The length of the TOS varied but was longer than in the X-subtype of the second pattern. Of the 52 analyzed hemispheres, two left and five right hemispheres harbored the fourth pattern type.

Images of all brains are displayed in the supplementary material (Supplementary Figure S2).

### Coordinate-Based Meta-analysis of Functional Imaging Studies Reporting Activations in the Lateral OFC Areas

**Figure 11:**
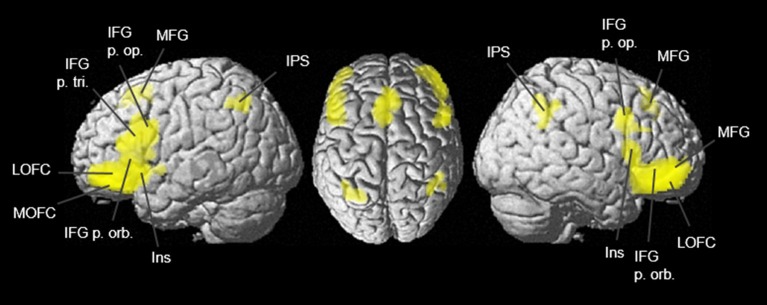
Conjunction analysis of lateral OFC areas Fo4–Fo7 was calculated with meta-analytic connectivity modeling (MACM) and projected onto the MNI ICBM 152 reference brain (Eickhoff et al., 2009). Conjunctional representations of combined co-activation of all four lateral OFC areas revealed joint co-activations in the dorsomedial and dorsolateral prefrontal cortex, the Broca region, and the intraparietal sulcus. Due to imaging modality, subcortical co-activations are not displayed although present.

With MACM, conjunctional analysis enabled the detection of all co-activational patterns in each lateral OFC area in both hemispheres (Figure 11). All lateral OFC areas showed co-activational connectivity with Broca’s region, the intraparietal sulcus, the dorsolateral prefrontal cortex as well as the Wernicke area. In addition, contrast analyses revealed differences in co-activation comparing two lateral OFC areas one at a time between the hemispheres (Supplementary Figure S4). All co-activation results were summarized in Supplementary Table S3.

Using the SPM Anatomy Toolbox (Eickhoff et al., 2005), co-activational clustering of the examined VOIs was connected with the cytoarchitectonically delineated areas of the JuBrain Cytoarchitectonic Atlas^5^ (Supplementary Tables S3, S4). The eight co-activational clusters of Fo4 represented co-activations with hIP1 and hIP3 (Choi et al., 2006) in both hemispheres, left area 2 (Grefkes et al., 2001), left and right area Fp1 (Bludau et al., 2014) as well as area Fo3 (Henssen et al., 2016), and left area 44 (Amunts et al., 2004). Fo5 showed co-activation with hIP1, hIP2, hIP3, areas 44 and 45, Fp1, Fo3 and area 2, each in both hemispheres and right inferior parietal area PFm (Caspers et al., 2006, 2008). Fo6 revealed co-activational patterns with left areas 44 and 45, as well as being strongly connected with hIP1, hIP2, hIP3, right area PFm and right area PGa (Caspers et al., 2006, 2008). Different from the other three lateral OFC areas, Fo7 showed co-activation with left area s32 (Vogt et al., 2013), left area Fp2 (Bludau et al., 2014), right area Fo3, left amygdala (laterobasal and centromedial nucleus) and the CA1 region of the left hippocampus as well as left area 45 and the left hippocampal amygdaloid transition area (Amunts et al., 2005).

The analysis of BDs and PCs resulted in different area-specific task-related functionalities based on the neuroimaging studies of the BrainMap database (Supplementary Figure S5 and Table 4). Hemispheric differences were detected in all four lateral OFC areas. Left Fo4 was involved in perceiving gustational input and taste. Left Fo5 had a strong bias towards reward processing and left Fo6 was activated during the processing of semantics in language-based tasks. Left Fo7 also showed activation in tasks concerning orthography, semantics, memory and gustational perception. The right Fo4 and Fo5 were co-activated while retrieving memory, right Fo5 and Fo6 share activities in the perception of touch, both comforting and painful. Also, activity while processing emotional behavior tasks was being observed in right Fo6, especially anger and disgust. Additionally, right Fo7 was active in face recognition tasks. Linking relationships between the four areas were also observed in other cognitive processes, e.g., areas Fo4, Fo5 and Fo7 were being activated in actions involving working memory. Areas Fo6 and Fo7 were active while conduction of tasks concerning attention as well as emotional induction.

**Table 4:** Functional characterization of lateral OFC areas for each hemisphere, adapted from the BDPC characterization of the MACM analysis (Eickhoff et al., 2009).

Area	Functional contribution
Fo4 left	Gustational perception Working memory
Fo4 right	Reward processing Working memory
Fo5 left	Reward processing Language processing: phonology Working and Explicit memory
Fo5 right	Explicit memory Perception of physical pain
Fo6 left	Language processing: semantics Disgust, Anger
Fo6 right	Perception of physical pain Action inhibition
Fo7 left	Gustational perception Olfactory perception Language processing: orthography Memory
Fo7 right	Emotional processing Face recognition

## Discussion

In the present study, four new cytoarchitectonic areas, Fo4, Fo5, Fo6, and Fo7, of the lateral OFC were identified, analyzed and mapped in 10 human postmortem brains. Their variable location and extent were reflected in the cytoarchitectonic probabilistic maps in two reference spaces, the MNI Colin27 space and the MNI ICBM 152 space. Co-activations and functional specifics of these areas showed area-specific functional connectivities with other brain regions. BDs and PCs showed lateralization towards a rational left-hemispheric lateral OFC and an emotional right-hemispheric lateral OFC.

The macroanatomy of the OFC revealed a variety of gyral and sulcal patterns. The medial and lateral orbital sulcus were connected through the TOS forming a bridge between the two former sulci. The TOS developed a distinct AOG and a visible separation of the posterior orbital gyrus from the other adjacent gyri. Several studies have identified similar shapes of sulci and gyri in the OFC and suggest a considerable interindividual variability. The *work by* Chiavaras and Petrides (2000) and Chiavaras et al. (2001) conducted an in-depth analysis of the orbital surface of the macaque and the human brain. Their work showed three different types of sulcal patterns based on the spatial variability in the OFC’s anatomy, which could be partially applied to the data of the present study. The ontological development of the brain changed the course of the gyri and sulci in a considerable way. The different patterns could be explained by various genetic and environmental factors. Chi et al. (1977) defined an increasing mediolateral and caudorostral trend of the OFC’s sulci, due to their varying chronology in the gestational formation. *According to Chi*, the lateral orbital sulcus is developing in the 32–35 weeks and the TOS later, at 36 weeks of gestation. All intermediate sulci emerge later at 40–44 weeks of gestation. Certain events, such as hereditary diseases caused by genetic mutations, metabolic disorders, environmental influences, etc., can influence the formation of the gyri and sulci in the fetal brain. *Chiavaras postulated* that sulci appearing early in gestation were more constant, whereas sulci appearing later were more variable (Chiavaras et al., 2001).

In addition, Rodrigues et al. (2015) have identified four different types of patterns of gyri and sulci in the OFC. Their patterning was supported by our discoveries, with the medial and lateral orbital sulcus being connected through a pronounced TOS or a complete separation of the sulci flowing into a less developed TOS. In contrast to Chiavaras and Petrides (2000), Rodrigues et al. (2015) extended their pattern detection by a fourth pattern, in which the lateral orbital sulcus was separated into rostral and caudal portions, but the medial orbital sulcus was longitudinally intact throughout the OFC region. Other authors also identified varying arrangements of sulci and gyri in the whole OFC. Earlier on, Ono et al. (1990) were able to find differing sulcal compositions. Inspecting the medial and lateral orbital sulcus, they postulated a division into anterior and posterior portions and found three different pattern types in the anterior parts of the OFC and four different types in the posterior OFC (Ono et al., 1990). More importantly, the number of sulci in the orbital region was also supported by our inspections. Ono et al. (1990) found three sulci ascending from the TOS which is analogous to our rostral portions of the medial and lateral orbital sulcus, as well as the intermediate orbital sulcus. In addition, two descending sulci were found posteriorly, which are equal to our caudal portions of the medial and lateral orbital sulcus. The classification of the sulci in the OFC also corresponded to that of the Automated Anatomical Labeling (AAL) atlas^6^, which was based on data from Chiavaras and Petrides (2000) and Chiavaras et al. (2001) and is now available online in its third version (Rolls et al., 2015). *Rolls postulated* that the AOG was inhabited by area 11 l (lateral), and the LOG by area 47/12 (Rolls et al., 2015). The former designations are based on Brodmann (1909), Öngür and Price (2000) and Öngür et al. (2003).

### Comparison to Previous OFC Maps and Methodical Limitations

von Economo and Koskinas (1925) delineated five areas in the OFC: F*Dp*, F*EF*, F*F*, F*Fa* and F*F*φ (Von Economo, 1929). Frontal areas F*Dp* and F*F*φ matched our delineated areas Fo5 and Fo6 in location and cytoarchitecture, respectively. The corresponding areal equivalent to area Fo4 was located in the F*EF* area. Furthermore, area F*F* extends topographically over the entire posterior orbital gyrus as well as the lateral orbital sulcus in which our Fo7 is located, but which does not exceed the TOS. Öngür and Price (2000) and Öngür et al. (2003) had identified four different areas in the lateral OFC, but just three matched our areas in location and structure, namely 47/12r in the rostral part of the lateral OFC corresponding to our area Fo5, the anterior part of 47/12 m analogous to our area Fo4, and 47/12l in the lateral parts of the ventral surface equivalent to our area Fo6. The reason for a missing locational or structural areal equivalent for area Fo7 was the fact that Öngür did not describe any areas hidden in the sulcal parts of the orbital surface. Nevertheless, their described laminar patterns were equal to our delineations of areas Fo4, Fo5 and Fo6. They found a sub laminated layer V and horizontal striations in layers III/IV in 47/12 m (Fo4), a granular layer IV in 47/12r (Fo5), and 47/12l (Fo6) being characterized by a stronger granular layer IV as well as large pyramidal cells in layer III, sharply demarcated layers and a sub laminated layer V (Öngür et al., 2003).

The identification of cytoarchitectonic areas of the lateral OFC was based on the Mahalanobis distance as a measure of cytoarchitectonic differences. Many studies of the past had identified boundaries between adjacent areas in a reproducible manner (Schleicher et al., 1998, 1999). It is less sensitive to gradual changes in the laminar pattern due to the integration of the covariance matrix changing at abrupt changes in cytoarchitecture between cortical areas (Schleicher et al., 1999; Morosan et al., 2001; Zilles et al., 2002). In order to consider differences in cortical geometry, the Mahalanobis distance was calculated for different block sizes of neighboring profiles (Schleicher et al., 1998). Borders were only accepted when the distance was significant for a large number of block sizes. Cortical regions, which were heavily tangentially cut, could not be analyzed by this method and were excluded (Amunts et al., 1999; Schleicher et al., 1999, 2000, 2005). Tangential sectioning of the cytoarchitecture represented a limitation to any type of analysis in 2D. However, alternative methods for cortical parcellation are under development, which use, for example, deep convolutional networks (Spitzer et al., 2018). In combination with high-resolution 3D models such as the Big Brain (Amunts et al., 2013), they open a new perspective to map the brain, independently on the angle of physical sectioning.

### Functional Connectivity of the Lateral OFC Areas

In agreement with previous studies (Elliott et al., 2000), areas in the lateral OFC showed co-activational connectivity with areas of the association cortex, also receiving gustatory and olfactory input as well as projecting to the central part of the caudate nucleus (Öngür and Price, 2000), hypothalamus (Rolls, 2000), hippocampus, amygdala and cingulate cortex (Kringelbach and Rolls, 2004; Ross et al., 2013). We were able to confirm these connections and extend them by cytoarchitectonically delineated areas from the frontal and parietal lobe and parts of the limbic system.

For areas Fo4 and Fo5, we found co-activations in Fp1 (Bludau et al., 2014), hIP1, hIP2 and hIP3 (Choi et al., 2006; Scheperjans et al., 2008a,b) in both hemispheres. These connectivities could explain the functional contribution of Fo4 and Fo5 in memory and reward processing. Fp1 (Bludau et al., 2014), which is involved in working memory, planning and cognition, is in close proximity to Fo4 and Fo5, probably linked with the anterior OFC areas through association fibers. Areas hIP1-3 (Choi et al., 2006; Scheperjans et al., 2008a,b) are also active during working memory, both spatial and object memory are being processed here. Functional connectivity of Fo4 to Fo3 (Henssen et al., 2016) could explain Fo4’s activity in gustational perception, possibly being a secondary gustational cortex region. The perception of pain with activity in the right areas Fo5 and Fo6 may also be explained by the functional connection to area PFm in the right inferior parietal lobule (Caspers et al., 2006, 2008). Attention reorientation, affective arousal and cognitive control were known functions of PFm and could explain the connection to right Fo5 and Fo6, which probably runs through the superior longitudinal fasciculus, since only the right PFm was activated. The pars orbitalis of the IFG is known to be activated in tasks which involve listening to music as well as in the processing of temporal coherence in music (Levitin and Menon, 2003). Semantics and musical structure tend to be related to each other (Levitin and Menon, 2003), what would explain the activations in the lateral OFC, especially area Fo6, in semantic tasks (Papathanassiou et al., 2000; Levitin and Menon, 2003; Amunts et al., 2004; Zald et al., 2014).

Additionally, areas 44 and 45 (Amunts et al., 2004) were co-activated with our areas Fo5, Fo6 and Fo7 in the left hemisphere, adding a language-processing role to them and broaden the knowledge of already known language-related cortical areas, as well as including a functional lateralization to the lateral OFC. Area 44 and 45 are known to be activated in verbal fluency tasks and left area 45 showed higher activation in semantic tasks (Amunts et al., 2004). According to Keller et al. (2009), it was assumed that the cytoarchitectonic differences and the different co-activation of areas 44 and 45 were also reflected in different functions. We can confirm this, although our areas of the lateral OFC are only partially different. We see similarities rather in areas that are not spatially adjacent. According to the hierarchical cluster analysis, Fo4 and Fo6 as well as Fo5 and Fo7 are structurally similar. We cannot confirm these similarities on the basis of their individual functions.

In contrast to the left hemispheric areas, right Fo5, Fo6 and Fo7 were more activated in emotion-driven cognitive processes, probably because of their connections to area s32 in the rectal gyrus (Vogt et al., 2013; Palomero-Gallagher et al., 2015), Fp2 (Bludau et al., 2014) in the medial frontal polar cortex, the hippocampus and the laterobasal and centromedial nuclei of the amygdala (Amunts et al., 2005). Area s32 is known to be activated in the processing of fear rather than sadness as well as in the processing of reward (Palomero-Gallagher et al., 2015), which corresponds to our activations in right Fo5. Area Fp2 was activated in social cognition and emotional processing (Bludau et al., 2014), which corresponded well with our functional discoveries for right areas Fo6 and Fo7. The hippocampus is well known in memory tasks (Amunts et al., 2005). Its CA1 region is activated in the processing of autobiographical and episodic memories among others (Bartsch et al., 2011), which was consistent with the functions of our right areas Fo5 and Fo6. Finally, the amygdala is mostly known to be involved in the processing of emotions, especially fear and fear conditioning (Sah et al., 2003). This connection gives the right area Fo6 more importance in the processing of fear and other negative emotions, such as anger. One possible connection between the amygdaloid nuclei and the orbital cortex could be the uncinated fasciculus, which was imaged and described elsewhere (Kier et al., 2004). The fasciculus extends into the superior, medial and inferior temporal gyrus as well as into the gyrus rectus, the medial and lateral orbital cortex and the orbital portion of the IFG (Kier et al., 2004). Possibly, the right Fo6 receives information with emotional content from the amygdaloid nuclei, which can then be analyzed to make decisions appropriate to the context. Actions may then be adapted accordingly. Also, a weak activation in the right pars orbitalis of the IFG was detected together with the adjacent anterior insula while listening to music (Levitin and Menon, 2003; Alluri et al., 2013). These activations do not need to be driven either by auditory or linguistic stimuli to process the temporal coherence of music (Levitin and Menon, 2003).

Our findings indicated that the anterior areas of the lateral OFC, areas Fo4 and Fo5, were both of importance in working and explicit memory, which already was reported in various studies (Rolls, 2000; Wallis, 2007; Rolls and Grabenhorst, 2008; Ross et al., 2013; Zald et al., 2014). Further, several studies reported the lateral OFC being activated in reward guided-behavior (Elliott et al., 2000; Rolls, 2000, 2004; Kringelbach and Rolls, 2004; Wallis, 2007; Rolls and Grabenhorst, 2008; Zald et al., 2014; Neubert et al., 2015; Cho et al., 2016; Dalton et al., 2016; Troiani et al., 2016; Rudebeck et al., 2017; Rudebeck and Rich, 2018), which could be corroborated in the present study for left area Fo5.

The lateral and posterior located areas Fo6 and Fo7 showed a more well-defined functional lateralization. Hence, processing language, especially semantics and orthography, in the left hemisphere with co-activations in the Broca region (Zald et al., 2014). The lateral OFC region is also activated in somatosensory and emotional processing, e.g., anger and disgust, in the right hemisphere with co-activations in the inferior parietal sulcus areas hIP1–hIP3 (Choi et al., 2006; Scheperjans et al., 2008a,b), as well as the amygdala, the hippocampus and the HATA region (Amunts et al., 2005). These observations were also reported in past studies (Kringelbach and Rolls, 2004; Nestor et al., 2013; Ross et al., 2013). According to Elliott et al. (2000), the right lateral OFC was associated with responses to angry faces but not to neutral faces, which corresponds well with our results with right area Fo6 being active while processing anger. All four examined lateral OFC areas seem to be involved in higher-order cognitive functions with a strong lateralization into rational task-based processing in the left hemisphere and the processing of emotionally charged behavior in the right hemisphere according to the results of the functional decoding (see also Supplementary Figure S5 and Table 4).

## Conclusion

The statistically reproducible cytoarchitectonical border detection in 10 postmortem brains allowed to identify four new cytoarchitectonically distinct areas Fo4, Fo5, Fo6, and Fo7. Probabilistic maps were computed demonstrating for the first time their different locational extent and interindividual variability. The functional meta-analysis of the lateral OFC assigned individual functions to its areas which revealed an interhemispheric lateralization. The processing of language and working memory revealed activations in the left hemisphere as opposed to the perception of gustational input, physical pain as well as emotional processing in the right hemisphere. The new maps of the areas in the lateral OFC were included in the publicly available JuBrain Cytoarchitectonic Atlas (DOI: 10.25493/8EGG-ZAR), furthermore they are available as separate downloads at the DOIs: 10.25493/29G0-66F (for Fo4), 10.25493/HJMY-ZZP (for Fo5), 10.25493/34Q4-H62 (for Fo6), and 10.25493/3WEV-561 (for Fo7). All maps are available in the MNI Colin27 and MNI ICBM 152 reference spaces. Since the lateral OFC was also natively mapped in the “BigBrain,” all maps are available in the “BigBrain” space as well. The fact that the maps are offered in different reference spaces and thus also in different resolutions enables a specific and adapted use by a broad readership. The here presented new insights of the lateral OFC may provide a better understanding of its functional relevance and the new maps can now be used as an anatomical reference for *in vivo* mapping procedures.

## Data Availability Statement

All datasets generated for this study are included in the article/Supplementary Material.

## Ethics Statement

Body donors gave written informed consent for the general use of postmortem tissue used in this study for aims of research and education. The usage is covered by a vote of the ethics committee of the medical faculty of the Heinrich Heine University Düsseldorf (#4863).

## Author Contributions

Cytoarchitectonic characterization, statistical border detection of the lateral OFC areas and statistics were performed and calculated by MW with support by KA and SC. KA designed the study. Prior lateral OFC analysis by FG was part of the analysis. Hierarchical cluster analysis was prepared by MW and calculated by SB. Volumetric analysis was estimated by HM and final calculations were made by MW and SB. Probabilistic maps and MPMs were calculated by HM, final adaptions of these maps were made by MW. Functional characterization was estimated by MW and MACM analysis was conducted by MW with support by SE. Discussion of results and writing were performed by all co-authors.

## Conflict of Interest

The authors declare that the research was conducted in the absence of any commercial or financial relationships that could be construed as a potential conflict of interest.

## Footnotes

^1^https://jubrain.fz-juelich.de/apps/cytoviewer/cytoviewer.php

^2^https://bigbrain.humanbrainproject.org/

^3^https://www.humanbrainproject.eu/en/explore-the-brain/atlases/

^4^http://brainmap.org

^5^https://jubrain.fz-juelich.de/apps/cytoviewer/cytoviewer.php

^6^http://www.gin.cnrs.fr/en/tools/aal/
