# Supplementary material for: Cytoarchitectonic Characterization and Functional Decoding of Four New Areas in the Human Lateral Orbitofrontal Cortex
Source: Front Neuroanat. 2020 Feb 5;14:2. doi: 10.3389/fnana.2020.00002 (PMC7014920; doi:10.3389/fnana.2020.00002)
Supplement: Supplementary file 1 [file Data_Sheet_S1.DOCX]

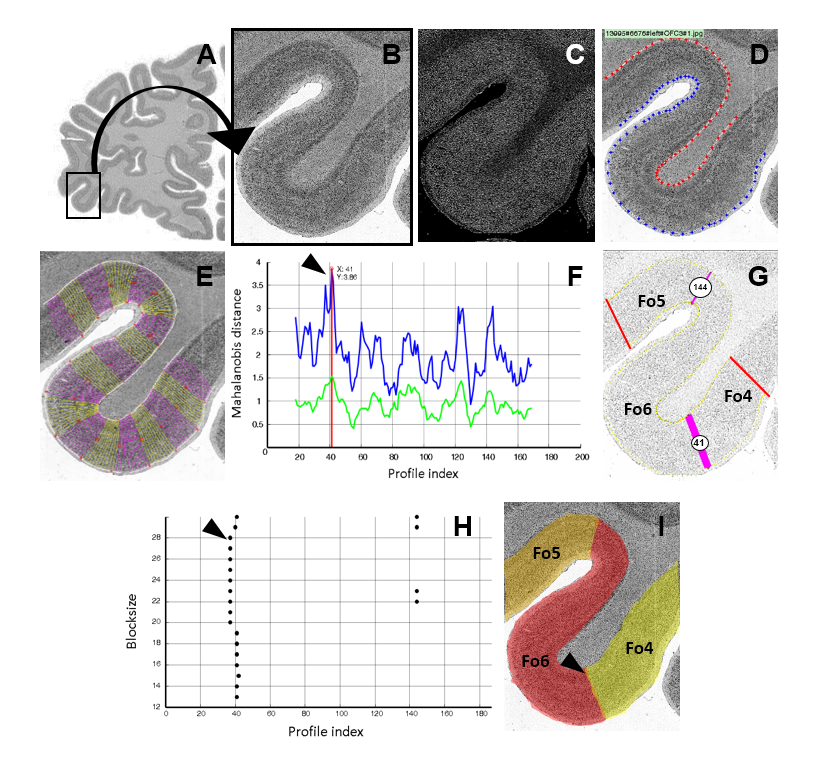


**Supplementary Figure 1.** Image sequence of statistically reproducible detection of cytoarchitectonical borders. A) digitized image of used brain section (left hemisphere) with region of interest (ROI) in the lateral orbital gyrus (black box), B) high-resolution scan of ROI in histological brain section, C) generated GLI-image, D) screenshot of drawn outer and inner contour lines encompassing ROI, E) curvilinear traverses running perpendicular to the cortex, F) plotted Mahalanobis distance (MD) calculation, G) verified border detection at MD’s highest outburst, H) dot plot of MD calculation over block sizes 12-30, I) detected areas drawn into digitized image.

**
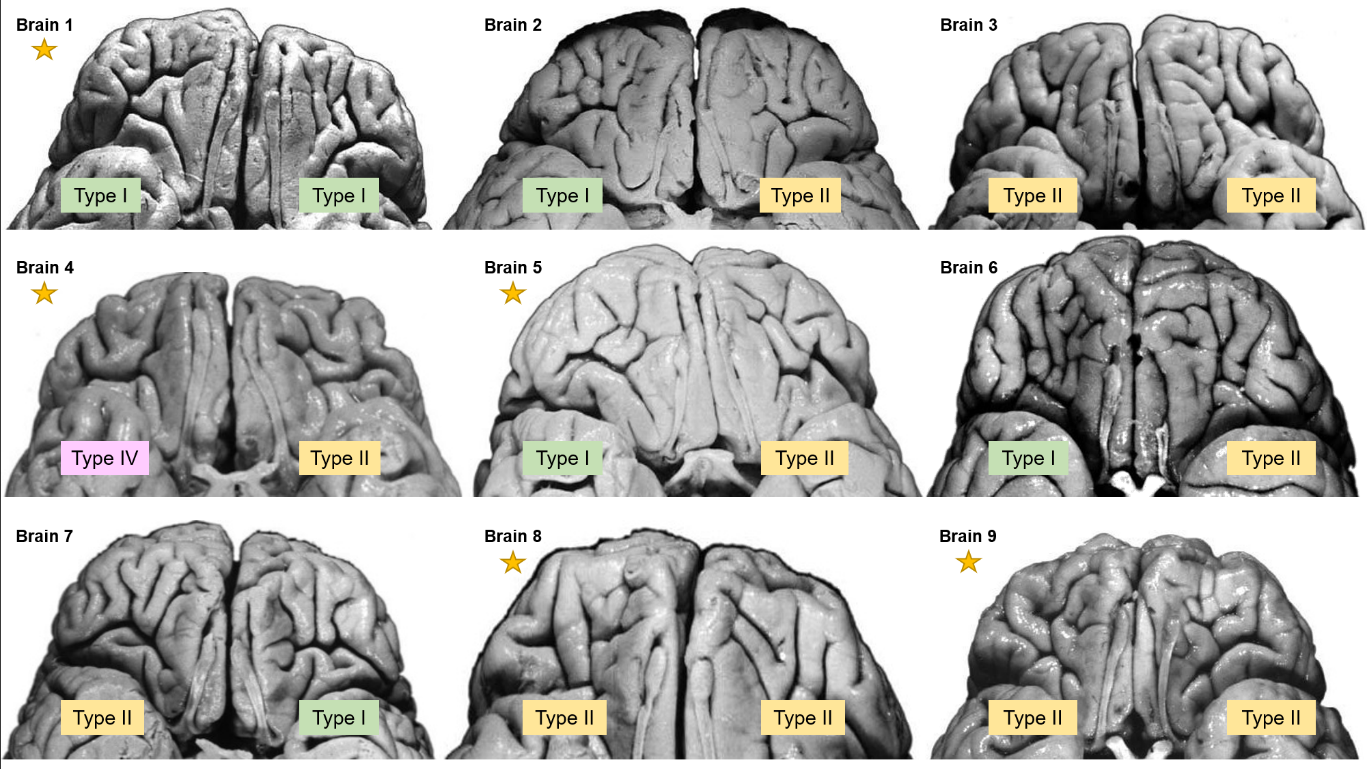

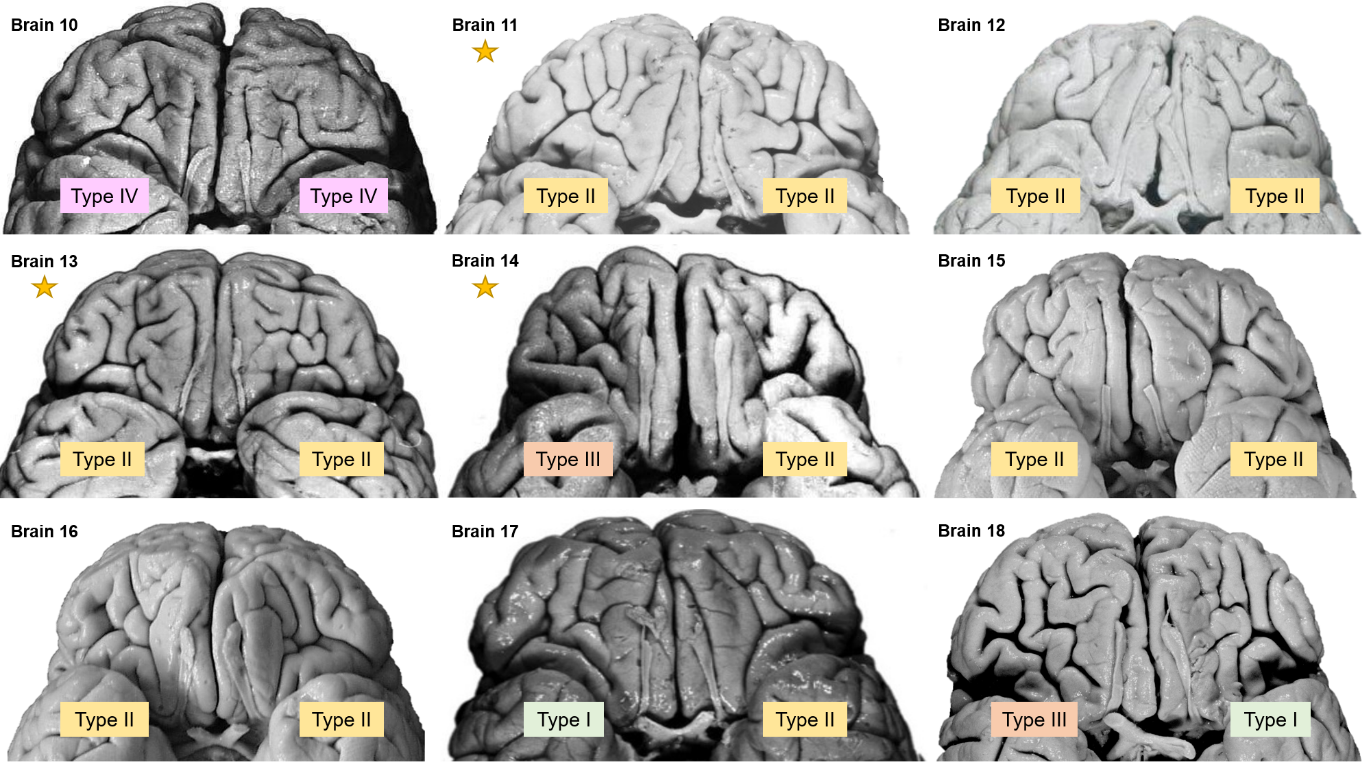

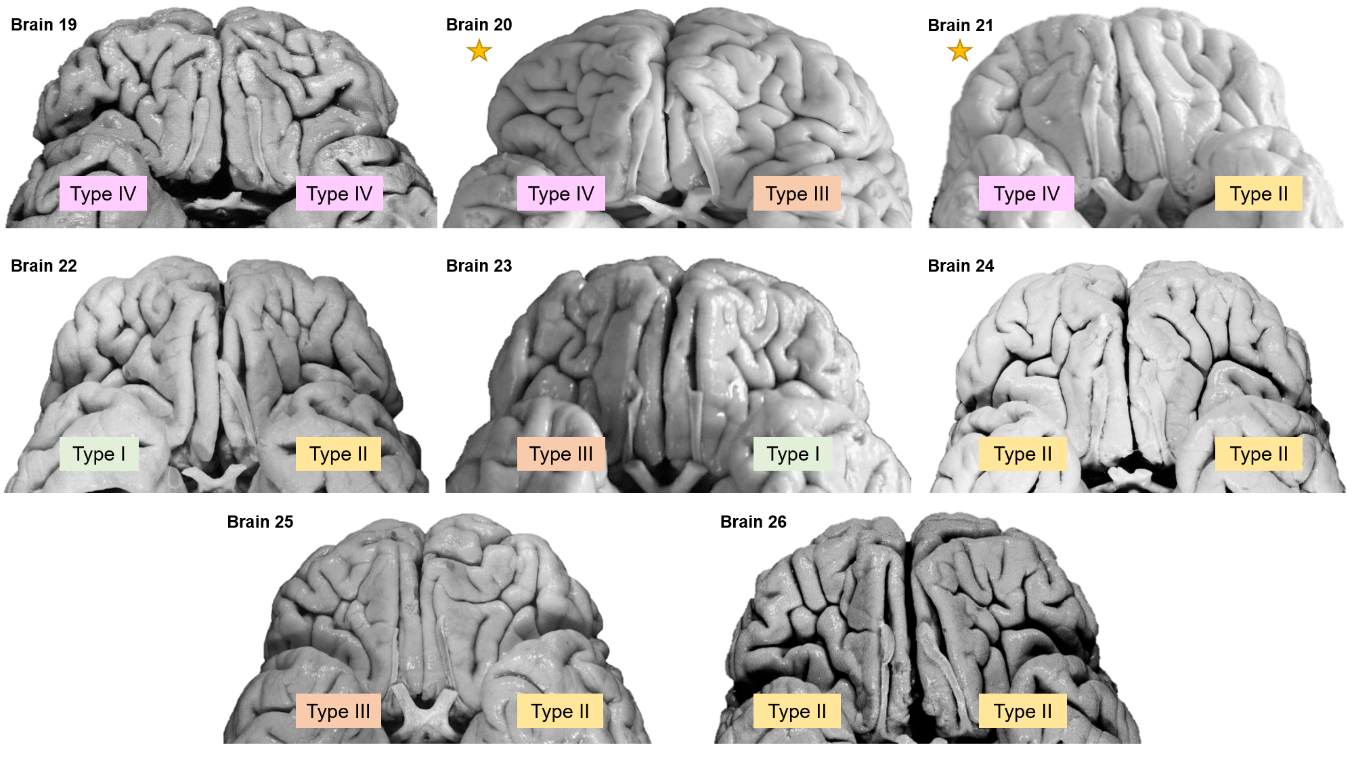
**

**Supplementary Figure 2.** All 26 brains were used for the macroanatomical pattern detection. For the display, the brain images were cut off at the level of the optic chiasm. Brains used for the cytoarchitectonic characterization are marked by a yellow star. Brain images were adapted from the JuBrain database.

**Supplementary Figure 3.** Classification of different patterns of sulci in the left and right hemisphere in a total number of 26 brains. Standard deviation is given by error indicators.


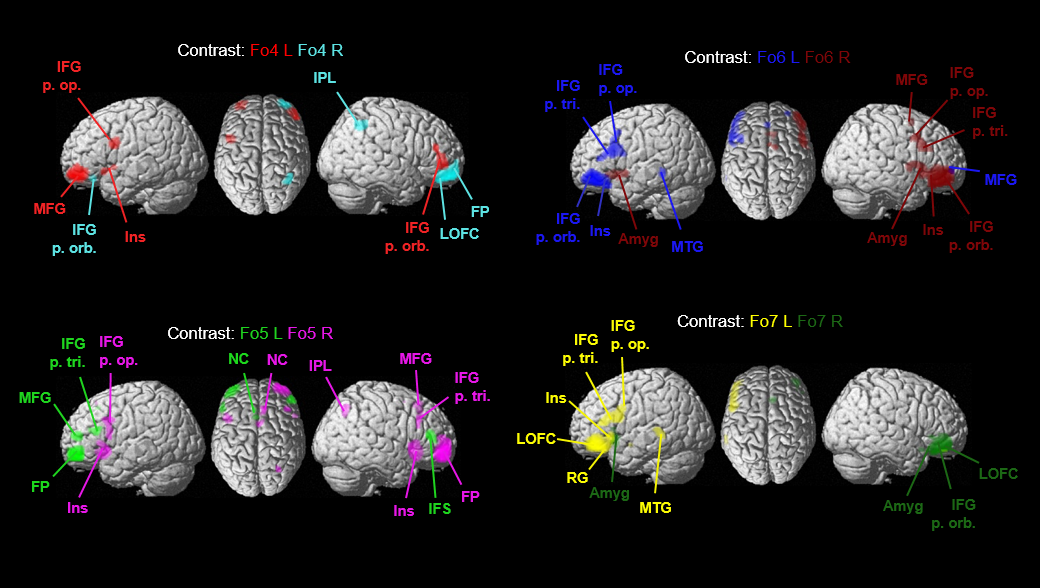


**Supplementary Figure 4.** Contrast analysis of lateral OFC areas Fo4 – Fo7 were calculated with MACM and projected onto the MNI ICBM 152 reference brain (Eickhoff et al., 2009). Each lateral OFC area contrast displayed area-specific co-activations in each hemisphere. Comparison of two areas revealed differences in co-activational and therefore functional connectivity. Due to imaging modality, subcortical co-activations are not displayed although present. L = left hemisphere, R = right hemisphere.


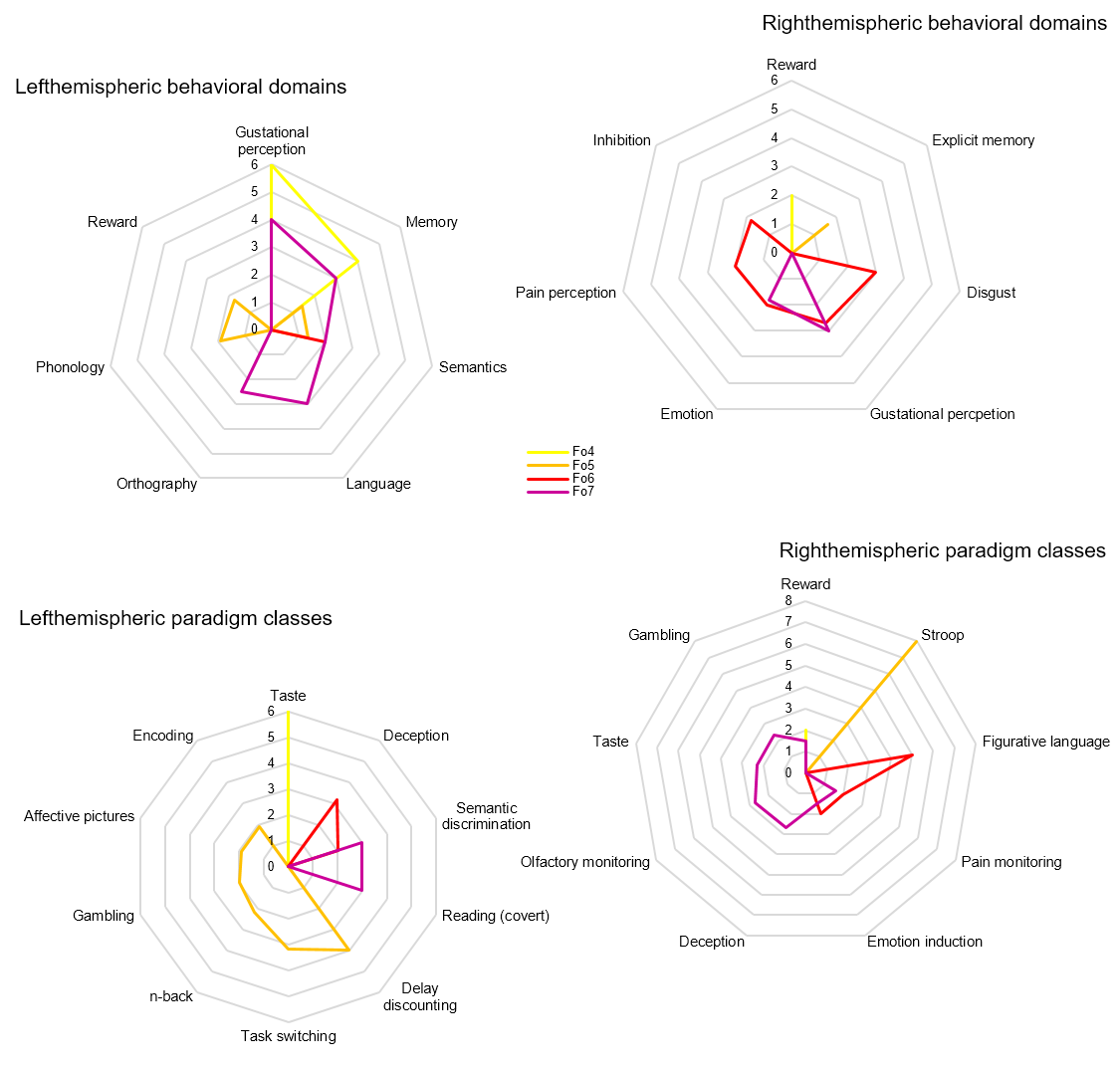


**Supplementary Figure 5.** Functional characterization of behavioral domains and paradigm classes in the lateral OFC areas Fo4 – Fo7 for the left and right hemispheres, adapted from the MACM analysis (Eickhoff et al., 2009). Results were considered significant at α = 0.05 (FDR-corrected). The color code of the respective areas corresponds to the colors in the MPM.
