# Supplementary material for: Cytoarchitectonic Characterization and Functional Decoding of Four New Areas in the Human Lateral Orbitofrontal Cortex
Source: Front Neuroanat. 2020 Feb 5;14:2. doi: 10.3389/fnana.2020.00002 (PMC7014920; doi:10.3389/fnana.2020.00002)
Supplement: Supplementary file 2 [file Table_S1.DOCX]

**Supplementary Table 1.** Sleuth search results of the BrainMap database (<http://www.brainmap.org>) (Fox et al., 2005; Fox & Lancaster, 2002). Merged maps were obtained by pooling left and right proportions of each areal maps. Therefore, results of pooled maps do not sum up as if adding results of left and right hemisphere together. 1,167 matching experiments were found with activations in the lateral OFC region.

| Area | Hemisphere | Found  papers | Subjects | Experiments w/  matching criteria | Conditions | Locations |
| --- | --- | --- | --- | --- | --- | --- |
| **Fo4** | Left | 129 | 1996 | 160 | 390 | 2335 |
|  | Right | 160 | 2315 | 187 | 417 | 2591 |
|  | Merged | 88 | 1467 | 107 | 222 | 1600 |
| **Fo5** | Left | 137 | 2135 | 158 | 345 | 2366 |
|  | Right | 232 | 3719 | 301 | 650 | 4403 |
|  | Merged | 113 | 1804 | 144 | 283 | 2245 |
| **Fo6** | Left | 247 | 3862 | 310 | 656 | 4384 |
|  | Right | 256 | 4173 | 324 | 672 | 4692 |
|  | Merged | 138 | 2290 | 171 | 370 | 2618 |
| **Fo7** | Left | 234 | 3758 | 288 | 609 | 3928 |
|  | Right | 246 | 3651 | 299 | 639 | 4127 |
|  | Merged | 200 | 3230 | 249 | 517 | 3305 |

**Supplementary Table 2.** Volumetric analysis of areas Fo4 - Fo7 of the lateral OFC per hemisphere in each of the ten postmortem brains.

| Brain | Areal volume [mm³] | | | | | | | | Sum |
| --- | --- | --- | --- | --- | --- | --- | --- | --- | --- |
|  | **Fo4 left** | **Fo4 right** | **Fo5 left** | **Fo5 right** | **Fo6 left** | **Fo6 right** | **Fo7 left** | **Fo7 right** |  |
| 1 | 538,74 | 818,85 | 1038,08 | 824,93 | 1886,73 | 1547,11 | 470,06 | 795,73 | **7920,23** |
| 2 | 655,49 | 801,53 | 553,19 | 330,26 | 833,24 | 1015,73 | 635,15 | 659,87 | **5484,45** |
| 3 | 438,85 | 384,01 | 341,68 | 511,70 | 938,36 | 737,28 | 695,29 | 565,21 | **4612,40** |
| 4 | 551,50 | 869,66 | 314,37 | 470,06 | 1772,18 | 1253,67 | 822,73 | 848,43 | **6902,60** |
| 5 | 641,99 | 905,03 | 401,30 | 519,75 | 1045,98 | 831,93 | 536,14 | 448,74 | **5330,86** |
| 6 | 713,02 | 947,93 | 672,47 | 668,71 | 1993,50 | 1643,25 | 710,45 | 642,41 | **7991,75** |
| 7 | 893,69 | 508,30 | 475,08 | 365,63 | 1145,09 | 1377,21 | 816,94 | 845,29 | **6427,23** |
| 8 | 605,70 | 730,04 | 235,66 | 798,19 | 1192,69 | 1012,71 | 638,33 | 366,95 | **5580,27** |
| 9 | 1020,97 | 780,77 | 797,29 | 608,79 | 959,67 | 920,93 | 325,48 | 604,88 | **6018,77** |
| 10 | 744,96 | 1174,73 | 362,02 | 674,66 | 1141,95 | 907,68 | 556,59 | 550,97 | **6113,56** |
| **Mean** | 680,49 | 792,08 | 519,11 | 577,27 | 1290,94 | 1124,75 | 620,72 | 632,85 | **6238,21** |
| **SD** | 173,02 | 221,44 | 250,69 | 167,96 | 426,46 | 312,08 | 154,41 | 161,80 | **1101,14** |

**Supplementary Table 3.** Co-activational clustering of lateral OFC areas. Only cytoarchitectonic areas with highest probabilities were displayed. Calculated with MACM and connected to the respective cytoarchitectonic areas of the JuBrain Atlas through the SPM Anatomy Toolbox (Eickhoff et al., 2005).

| Cluster No. | Cluster size [Voxel] | Macroanatomical location | Cytoarchitectonic areas [probability] | MNI coordinates | | |
| --- | --- | --- | --- | --- | --- | --- |
|  |  |  |  | **X** | **Y** | **Z** |
| Co-activations Fo4 | | | | | | |
| 1 | 1302 | Right middle frontal gyrus | Right area Fp1 [20.4%] | 34 | 54 | 12 |
|  |  | Right middle orbital gyrus | Right area Fo3 [11.3%] | 32 | 50 | -16 |
| 2 | 1007 | Left middle orbital gyrus | Left were Fp1 [25.9%] | -30 | 52 | -14 |
|  |  |  | Left area Fo3 [14.0%] |  |  |  |
| 3 | 764 | Left inferior frontal gyrus (pars triangularis) | Left area 45 [1.0%] | -40 | 36 | 8 |
|  |  |  | Left area 44 [0.3%] | -48 | 22 | 20 |
|  |  | Left insula lobe |  | -32 | 20 | -4 |
| 4 | 698 | Left midcingulate cortex |  | 0 | 26 | 34 |
| 5 | 648 | Left inferior parietal lobule | Left area hIP3 (IPS) [37.7%] | -36 | -52 | 44 |
|  |  | Left superior parietal lobule | Left area hIP1 (IPS) [43.1%] | -28 | -60 | 48 |
|  |  | Left inferior parietal lobule | Left area 2 [6.7%] | -42 | -40 | 48 |
| 6 | 528 | Left precentral gyrus | Left area 44 [23.6%] | -50 | 10 | 34 |
| 7 | 335 | Right inferior parietal lobule | Right area hIP3 (IPS) [44.1%] | 34 | -54 | 46 |
|  |  |  | Right area hIP1 [32.3%] |  |  |  |
| 8 | 261 | Right inferior frontal gyrus (pars orbitalis) |  | 34 | 24 | -10 |
| Co-activations Fo5 | | | | | | |
| 1 | 2559 | Right middle orbital gyrus | Right area Fp1 [15.9%] | 34 | 58 | -4 |
|  |  | Right inferior frontal gyrus (pars triangularis) | Right area 45 [6.9%] | 50 | 24 | 28 |
| 2 | 1273 | Left precentral gyrus | Left area 44 [15.3%] | -46 | 8 | 30 |
|  |  | Left inferior frontal gyrus (pars triangularis) | Left area 45 [9.6%] | -46 | 26 | 20 |
| 3 | 1110 | Left superior medial gyrus |  | 0 | 26 | 40 |
|  |  | Left posterior medial frontal gyrus |  | 2 | 22 | 48 |
| 4 | 1078 | Left middle orbital gyrus | Left area Fp1 [19.4%] | -36 | 56 | -8 |
| 5 | 812 | Left inferior parietal lobule | Left area hIP3 (IPS) [29.5%] | -36 | -48 | 48 |
|  |  |  | Left area hIP1 (IPS) [14.4%] |  |  |  |
|  |  |  | Left area 2 [11.4%] | -48 | -36 | 48 |
|  |  |  | Left area hIP2 (IPS) [8.9%] |  |  |  |
| 6 | 735 | Right inferior frontal gyrus (pars opercularis) | Right area 44 [2.2%] | 52 | 18 | -2 |
|  |  |  | Right area 45 [1.4%] |  |  |  |
| 7 | 666 | Left insula lobe |  | -32 | 20 | -4 |
| 8 | 572 | Right angular gyrus | Right area hIP3 (IPS) [25.5%] | 38 | -60 | 50 |
|  |  | Right inferior parietal lobule | Right area hIP2 (IPS) [21.6%] | 48 | -44 | 48 |
|  |  |  | Right area PFm (IPL) [14.6%] |  |  |  |
|  |  | Right angular gyrus | Right area hIP1 (IPS) [10.4%] | 44 | -58 | 36 |
| 9 | 208 | Right caudate nucleus |  | 14 | 14 | -6 |
| 10 | 199 | Left caudate nucleus |  | -14 | 6 | 10 |
| Co-activations Fo6 | | | | | | |
| 1 | 3065 | Left inferior frontal gyrus (pars triangularis) | Left area 45 [10.4%] | -50 | 20 | 22 |
|  |  | Left inferior frontal gyrus (pars opercularis) | Left area 44 [8.0%] | -48 | 10 | 28 |
| 2 | 2264 | Right inferior frontal gyrus (pars opercularis) | Right area 44 [5.6%] | 54 | 16 | 10 |
|  |  |  | Right area 45 [4.4%] |  |  |  |
| 3 | 1166 | Left superior medial gyrus |  | -4 | 26 | 40 |
| 4 | 251 | Right inferior frontal gyrus (pars triangularis) | Right area 45 [5.2%] | 52 | 24 | 26 |
| 5 | 219 | Left superior parietal lobule |  | -26 | -62 | 48 |
|  |  | Left inferior parietal lobule | Left area hIP3 (IPS) [52.7%] | -36 | -60 | 44 |
|  |  |  | Left area hiP1 (IPS) [20.4%] |  |  |  |
| 6 | 194 | Right inferior parietal lobule | Right area hIP1 [IPS) [48.8%] | 42 | -50 | 40 |
|  |  |  | Right area hIP2 (IPS) [11.7%] | 50 | -42 | 46 |
|  |  |  | Right area PFm (IPL) [7.0%] |  |  |  |
|  |  | Right angular gyrus | Right area PGa (IPL) [9.4%] | 52 | -52 | 30 |
| Co-activations Fo7 | | | | | | |
| 1 | 3447 | Left inferior frontal gyrus (pars triangularis) | Left area 45 [8.3%] | -46 | 30 | 16 |
| 2 | 1593 | Right inferior frontal gyrus (pars orbitalis) | Right area Fo3 [13.7%] | 36 | 36 | -16 |
| 3 | 808 | Left superior medial gyrus |  | -4 | 24 | 40 |
|  |  | Left posterior medial frontal gyrus |  | 0 | 16 | 58 |
| 4 | 267 | Left rectal gyrus | Left area s32 [20.4%] | -2 | 42 | -16 |
|  |  |  | Left area Fp2 [17.0%] |  |  |  |
| 5 | 217 | Left middle temporal gyrus | Left area TE3 [0.5%] | -56 | -40 | 6 |
| 6 | 139 | Left hippocampus | Left amygdala (LB) [21.1%] | -22 | -10 | -20 |
|  |  |  | Left amygdala (CM) [13.8%] |  |  |  |
|  |  |  | Left hippocampus (CA1) [9.7%] |  |  |  |
|  |  |  | Left HATA region [4.4%] |  |  |  |

**Supplementary Table 4.** Co-activated cortical areas and subcortical nuclei and their reported functional representations according to cited references.

| Coactivated area/nucleus | Functions | Reference |
| --- | --- | --- |
| **2** | Shape and curvature changes, higher level of somatosensory processing | Grefkes, Geyer, Schormann, Roland, & Zilles, 2001 |
| **44** | Language production & perception, phonologic & syntax processing, verbal fluency, spontaneous speech, semantic processing, word production | Amunts et al., 2004 |
| **45** | Language production & perception, phonologic & syntax processing, verbal fluency, spontaneous speech, semantic processing, word production | Amunts et al., 2004 |
| **amygdala CM** | Defense, escape, pain, motivation, emotional discrimination | Amunts et al., 2005 |
| **amygdala LB** | Defense, escape, pain, motivation, emotional discrimination | Amunts et al., 2005 |
| **CA1** | Learning, memory | Amunts et al., 2005 |
| **Fo3** | Left: hedonic judgements of odor stimuli, emotional rating of visual & auditory stimuli;  Right: familiarity judgements;  Whole: expectation of pleasant taste, positive memories | Henssen et al., 2016 |
| **Fp1** | Cognition, episodic & working memory, perception, planning | Bludau et al., 2014 |
| **Fp2** | Affective processing, social cognition, emotional processing | Bludau et al., 2014 |
| **HATA** | Defense, escape, pain, motivation, emotional discrimination, learning, memory | Amunts et al., 2005 |
| **hIP1** | Numerical processing, visuo-spatial & visuo-motor operations, spatial & object working memory | Choi et al., 2006 |
| **hIP2** | Numerical processing, calculation | Choi et al., 2006 |
| **hIP3** | Visuo-motor integration, reaching movements, calculation | Scheperjans, Eickhoff, et al., 2008; Scheperjans, Hermann, et al., 2008 |
| **PFm** | Right: attention reorientation, affective arousal, movement planning, cognitive control | Caspers et al., 2008, 2006 |
| **PGa** | Language-related processing, semantics, phonological processing | Caspers et al., 2008, 2006 |
| **s32** | Negative emotions, value-dependent changes for monetary reward, physical pain, autonomic functions, emotional memories | Vogt et al., 2013 |
